# Sexual dysfunction among married couples living in Kumasi metropolis, Ghana

**DOI:** 10.1186/1471-2490-11-3

**Published:** 2011-03-02

**Authors:** Nafiu Amidu, William KBA Owiredu, Christian K Gyasi-Sarpong, Eric Woode, Lawrence Quaye

**Affiliations:** 1 Department of Medical Laboratory Technology, Faculty of Allied Health Sciences, College of Health Sciences, Kwame Nkrumah University of Science and Technology, Kumasi, Ghana; 2 Department of Molecular Medicine, School of Medical Sciences, College of Health Sciences, Kwame Nkrumah University of Science and Technology, Kumasi, Ghana; 3 Department of Surgery, (Urology Unit) Komfo Anokye Teaching Hospital/ College of Health Sciences, Kwame Nkrumah University of Science and Technology, Kumasi, Ghana; 4 Department of Pharmacology, Faculty of Pharmacy and Pharmaceutical Science, College of Health Sciences, Kwame Nkrumah University of Science and Technology, Kumasi, Ghana

## Abstract

**Background:**

Sexuality and its manifestation constitute some of the most complex of human behaviour and its disorders are encountered in community. Sexual dysfunction is more prevalent in women than in men. While studies examining sexual dysfunction among males and females in Ghana exist, there are no studies relating sexual problems in males and females as dyadic units. This study therefore investigated the prevalence and type of sexual disorders among married couples.

**Method:**

The study participants consisted of married couples between the ages of 19 and 66 living in the province of Kumasi, Ghana. Socio-demographic information and Golombok-Rust Inventory of Sexual Satisfaction (GRISS) questionnaires were administered to 200 couples who consented to take part in the study. All 28 questions of the GRISS are answered on a five-point (Likert type) scale from "always", through "usually', "sometimes", and "hardly ever", to "never". Responses are summed up to give a total raw score ranging from 28-140. The total score and subscale scores are transformed using a standard nine point scale, with high scores indicating greater problems. Scores of five or more are considered to indicate SD. The study was conducted between July and September 2010.

**Results:**

Out of a total of 200 married couples, 179 completed their questionnaires resulting in a response rate of 89.5%. The mean age of the participating couples as well as the mean duration of marriage was 34.8 ± 8.6 years and 7.8 ± 7.6 years respectively. The husbands (37.1 ± 8.6) were significantly older (p < 0.0001) than their corresponding wives (32.5 ± 7.9). After adjusting for age, 13-18 years of marriage life poses about 10 times significant risk of developing SD compared to 1-6 years of married life among the wives (OR: 10.8; CI: 1.1 - 49.1; p = 0.04). The total scores (6.0) as well as the percentage above the cut-off (59.2) obtained by the husbands compared to the total score (6.2) and the percentage above cut-off (61.5) obtained by the wives, indicates the likely presence of sexual dysfunction. The prevalence of impotence and premature ejaculation were 60.9% and 65.4% respectively from this study and the prevalence of vaginismus and anorgasmia were 69.3% and 74.9% respectively. The highest prevalence of SD subscales among the men was dissatisfaction with sexual act followed by infrequency, whereas the highest among the women was infrequency followed by anorgasmia. Dissatisfaction with sexual intercourse among men correlated positively with anorgasmia and wife's non-sensuality and infrequency of sex.

**Conclusion:**

The prevalence of sexual dysfunction in married couples is comparable to prevalence rates in the general male and female population and is further worsened by duration of marriage. This could impact significantly on a couple's self-esteem and overall quality of life.

## Background

Marriage, a union between men and women is intended as a source of happiness, pleasure, compassion, love, a powerful form of communication and it typically provides a reliable means for satisfaction of sexual desires [1]. The success of a marriage largely depends on the sexual relationship between the couple. Ignorance of the sexual need of each other has led to many broken homes as well as extra-marital affairs [1]. Inability to perform up to the partner's expectation in a sexual relationship is usually considered as a weakness. Marthol and Hilz, [2] defined sexual dysfunction as the disturbances in sexual desire and the psycho-physiological changes that characterize the sexual response and cause marked distress and interpersonal difficulty. SD is a mixture of problems that has both biologic and psychosocial components and is multi-factorial in terms of aetiology. SD is an important public health problem that is more prevalent in women [3] than in men [4].

How a man would react to his wife's SD may depend on his psychological and sexual susceptibility. Inability to penetrate his wife due to her SD might lead to frustration or feeling of rejection [5]. A normal sexually functional man may develop secondary impotence in response to their wives' disorder [5].

Factors such as stress, negative emotional response, anxiety, sexual dissatisfaction and infrequency of intercourse are associated with happiness in marriage [6]. Frank *et al.*, [7], Schenk *et al.*, [8] and Lawrance *et al.*, [9] also indicated that the level of sexual dissatisfaction had a negative impact on the quality of the relationship between the couples. It has been suggested that satisfaction with the sexual relationship plays a vital role in creating and maintaining a happy marriage [6,10-14]. However, this relation between marital sexual satisfaction and SD has been reported to be stronger in men than in women [15]. Rust *et al.*, [15] indicated that premature ejaculation and erectile dysfunction had a stronger relation with marital unhappiness than vaginismus and orgasm disorders in women. Whereas SD is usually perceived to be an indication of disturbed relations between spouses by family and marital therapists, sex therapists are more likely to focus on the SD in the therapeutic context. The level of non-communication and affection between marriage couples, expression of feelings and thoughts which are extremely important to a couple's marital satisfaction are usually ignored.

Generally, there is paucity of data among Africans concerning sexual dysfunction probably because of the conservative nature of most African cultures. Previous reports among Ghanaians indicated the prevalence of sexual dysfunction to be 66% among males [4], 73% among females [3] and 60% among men presenting with various medical conditions [16]. However, very little research if at all has examined the prevalence of SD and its subscales among African married couples as dyadic units. To date, no study has examined how such central elements of marital functioning might influence each other when predicting marital sexual satisfaction and as such the sole purpose of this study is to address such gaps.

## Methods

### Participants

This epidemiological cross-sectional study was conducted among community dwellers in the Kumasi metropolis, Ghana between July and September 2010. Questionnaires were administered to a total of 200 heterosexual couples. Eligibility criteria for participants were as follows: heterosexual couples, aged 18 years or older who are in good health with marriage duration of not less than 6 months. The age range of the men involved was between 20 and 66 years, whereas the age range of their corresponding wives was between 19 and 58 years. The duration of marriage ranges from 6 months to 35 years. Participation of the respondents was voluntary and informed consent was obtained from each participant. The study was approved by the Committee on Human Research, Publication and Ethics of the School of Medical Science and the Komfo Anokye Teaching Hospital, Kumasi.

### Procedure

All couples were evaluated by using a semi-structured questionnaire and the Golombok Rust Inventory of Sexual Satisfaction (GRISS).

### Questionnaire

The questionnaire was used to determine the socio-demographic variables such as age, gender, duration of marriage, education level, smoking, alcohol consumption and exercise level. Socio-demographic variables such as age, marital status, years of scholarship, smoking status, level of exercise and alcohol intake were recorded. Exercise was defined as any activity causing light perspiration or a slight to moderate increase in breathing or heart rate for at least 30 minutes. Alcohol intake was defined as the intake of at least one bottle of an alcoholic beverage per week. Regarding smoking, individuals were classified as smokers based on whether the respondent is in the habit of smoking at least one cigarette a day. High education was defined as having attained at least tertiary education.

### The Golombok Rust Inventory of Sexual Satisfaction

Sexual response was measured by the Golombok Rust Inventory of Sexual Satisfaction (GRISS) questionnaire. The GRISS, in separate forms for men and women, has 28 items on a single sheet and is used for assessing the existence and severity of sexual problems in heterosexual couples or individuals who have a current heterosexual relationship. All the 28 questions are answered on a five-point (Likert type) scale from "always", through "usually', "sometimes", and "hardly ever", to "never". It provides overall scores, for men and women separately, of the quality of sexual functioning within a relationship. In addition, subscale scores of impotence, premature ejaculation, anorgasmia, vaginismus, infrequency, non-communication, male dissatisfaction, female dissatisfaction, male non-sensuality, female non-sensuality, male avoidance and female avoidance can be obtained and represented as a profile. Responses are summed up to give a total raw score (range 28-140). The total score and subscale scores are transformed using a standard nine point scale, with high scores indicating greater problems. Scores of five or more are considered to indicate SD [17]. The GRISS was chosen because it is standardized, easy to administer and score, relatively unobtrusive and substantially inexpensive.

The GRISS can be used to assess improvement as a result of sexual or marital therapy and to compare the efficacy of different treatment methods. It can also be used to investigate the relationship between sexual dysfunction and extraneous variables. The subscales are particularly helpful in providing a profile for diagnosis of the pattern of sexual functioning within the couple, which can be of great benefit in designing a treatment program. The reliability of the overall scales has been found to be 0.94 for men, 0.87 for women and that of the subscales on average 0.74 (ranging between 0.61 and 0.83). Validity has been demonstrated under a variety of circumstances [17-19].

### Statistical analysis

The data were presented as mean ± SD or percentages. Logistic regression was used to assess the influence of different variables in sexuality. In all statistical tests, a value of *p*< 0.05 was considered significant. All analysis were performed using SigmaPlot for Windows, Version 11.0, (Systat Software, Inc. Germany) [20].

## Results

### Response rate and socio-demographic characteristic

Out of a total of 200 married couples approached, 179 completed their questionnaires. Twelve couples had either the wife or husband refusing to take part in the study, 2 couples had difficulties in understanding the questionnaires and the questionnaires from 7 couples were incomplete, indicating a response rate of 89.5%. The mean age of the participating couples as well as the mean duration of marriage was 34.8 ± 8.6 years and 7.8 ± 7.6 years respectively. The husbands (37.1 ± 8.6) were significantly older (p < 0.0001) than their corresponding wives (32.5 ± 7.9). Whereas 77.8% of the husbands had attained high education, 54.6% consumed alcoholic beverages, 29.5% were physically inactive (i.e. sedentary lifestyle) with only 7.8% being smokers. Also, 61.0% of the wives have attained high education, 50.3% were physically inactive, 27.0% consumed alcoholic beverages and only 1.7% smoked cigarettes. The proportion of husbands who have attained high education (p = 0.0008), smoked cigarettes (p = 0.0108) and consumed alcoholic beverages (p < 0.0001) were significantly higher compared to their corresponding wives except for physical inactivity which was significantly higher (p < 0.0001) for the wives compared to their corresponding husbands.

### Risk factors

The effects of different socio-demographic variables on SD risk among the married couples are recorded in Table 1. The only significant factor from this study that increased the wives SD as determined by univariate analysis was marriage, with 13-18 years of marriage posing about 6 times the risk of developing SD as compared to 1-6 years of marriage life (OR: 6.3; CI: 1.0 - 51.8; p = 0.04). However, husbands who have been married for > 18 years are about 3 times at risk of developing SD as compared to those who have been married for 1-6 years (OR: 2.7; CI: 1.0 - 7.8; p = 0.04). None of the other factors modified SD risk significantly (Table 1). After adjusting for age, 13-18 years of marriage life poses about 10 times risk of developing SD compared to 1-6 years of marriage life among the wives (OR: 10.8; CI: 1.1 - 49.1; p = 0.04). However, the association of marriage duration resolves for men after adjustment for age.

**Table 1:** Logistic regression analysis of the risk factors for sexual dysfunction stratified by spousal gender

	Husbands					Wives				
**Variables**	**SD(%)**	**OR(95% CI)**	**P value**	**aOR(95% CI)**	**P value**	**SD(%)**	**OR(95% CI)**	**P value**	**aOR(95% CI)**	**P value**

**Duration of marriage (yrs)**										
< 1	87.5	5.8(0.7-49.0)	0.1	5.6(0.6-47.7)	0.1	50.0	0.9(0.2-4.4)	0.9	0.7(0.2-3.6)	0.7
1-6*	51.2					56.1				
7-12	70.0	0.9(0.4-1.8)	0.7	0.9(0.4-2.2)	0.9	90.0	0.9(0.4-1.9)	0.8	1.2(0.5-2.8)	0.7
13-18	23.8	1.9(0.5-7.9)	0.4	2.2(0.5-10.6)	0.3	66.7	6.3(1.0-51.8)	0.04	10.8(1.1-49.1)	0.04
> 18	56.7	2.7(1.0-7.8)	0.04	3.5(0.5-23.1)	0.2	59.8	1.6(0.6-4.6)	0.4	4.5(0.6-33.2)	0.1
**Educational level**										
Low	64.1	1.3(0.6-2.7)	0.5	1.3(0.6-2.7)	0.5	66.7	1.4(0.8-2.7)	0.3	1.4(0.7-2.6)	0.3
High*	57.7					58.2				
**Smoking**										
Yes	50.0	0.7(0.2-2.0)	0.5	0.6(0.2-1.8)	0.3	33.3	0.3(0.0-3.4)	0.3	0.3(0.0-3.0)	0.3
No*	60.0					62.3				
**Alcohol consumption**										
Yes	64.2	1.6(0.9-2.9)	0.1	1.5(0.8-2.8)	0.2	66.7	1.3(0.7-2.7)	0.4	1.4(0.8-2.8)	0.4
No*	53.2					60.0				
**Exercise**										
Yes	61.3					57.5				
No*	51.9	0.7(0.3-1.3)	0.2	0.6(0.3-1.2)	0.2	65.2	1.4(0.8-2.6)	0.3	1.4(0.8-2.5)	0.3

*Reference group, SD = sexual dysfunction, OR = odds ratio, aOR = age adjusted odds ratio, CI = confidence interval

### Sexual function-GRISS

Table 2 presents the scaled score data obtained from the GRISS questionnaires. The total scores (6.0) as well as the percentage above the cut-off (59.2) obtained by the husbands as subjects were slightly lower than the total score (6.2) and the percentage above cut-off (61.5) when the wives were used as the subjects, indicating the likely presence of sexual dysfunction. However, the proportion of the female spouse with SD whose husband also had SD was 69.8% compared to a proportion 67.3% of the male spouse with SD whose wives also had SD.

**Table 2:** Incidence of sexual dysfunction and its subscale using GRISS among married couples (N = 179)

		Husbands subjects		Female spouse			Wives subjects		Male spouse	
**Variables**	**n**	**mean(Sd)**	**Dif**	**mean(Sd)**	Dif*	**n**	**mean(Sd)**	**Dif**	**mean(Sd)**	Dif*

Sexual dysfunction	106	6.0(1.2)	59.2	5.5(1.6)	69.8	110	6.2(1.0)	61.5	5.2(1.6)	67.3
Impotence	109	6.2(1.1)	60.9			110			5.2(2.0)	67.3
Premature ejaculation	117	6.2(1.0)	65.4			110			5.0(1.9)	64.5
Vaginismus	106			5.4(1.8)	76.4	124	6.1(1.1)	69.3		
Anorgasmia	106			5.4(1.4)	84.9	134	5.7(1.0)	74.9		
Avoidance	106	6.2(1.3)	59.2	5.5(1.8)	66.0	97	6.4(1.3)	54.2	5.6(1.8)	72.2
Non-sensuality	107	5.9(1.4)	59.8	5.5(1.7)	75.7	117	6.1(1.2)	65.4	5.1(1.7)	69.2
Non-communication	100	6.2(1.5)	55.9	5.1(1.9)	62.0	97	6.1(1.5)	54.2	5.0(1.8)	63.9
Dissatisfaction	148	6.8(1.4)	82.6	5.1(1.8)	65.5	109	6.0(1.3)	60.9	6.5(1.9)	89.0
Infrequency	136	5.8(1.1)	76.0	5.4(1.8)	85.3	137	6.0(1.2)	76.5	5.3(1.6)	84.8

Sd = standard deviation, Dif = percentage with sexual difficulties (i.e. score of 5 to 9), Dif* of the spouse (i.e. male and female spouse) is the percentage of the subjects (i.e. male and female subjects) with Dif who also have Dif.

Of the individual GRISS subscales, infrequency of sex was high in all cases. Male partners had lower rate of difficulties in most of the subscales, with the exception of dissatisfaction, avoidance and to some extent non-communication. Whereas the prevalence of impotence and premature ejaculation were 60.9% and 65.4% respectively from this study, the prevalence of vaginismus and anorgasmia were 69.3% and 74.9% respectively. However, about 76% and 85% of the wives whose husbands had SD also had vaginismus and anorgasmia respectively and about 67% and 65% of the husbands whose wives had SD also had impotence and premature ejaculation respectively (Table 2). The highest prevalence of SD subscales among the husbands was dissatisfaction with sexual act followed by infrequency whereas the highest among the wives was infrequency followed by anorgasmia. Interestingly, 66% of the wives of men who are not satisfied with their sexual performance were also dissatisfied in contrast to 89% of the husbands of the women who are not satisfied with their sexual acts. This trend is the same for avoidance of sex and non-communication but opposite for non-sensuality as shown in table 2.

### Relationships between variables

Within couples, SD in the husband correlated positively with a medium size effect with that of their spouse (r = 0.42, p < 0.001). For the purpose of interpretation, Cohen [21] considered 0.10 < r < 0.30 as small, 0.30 <*r *< 0.50 as medium and r > 0.50 as large. Wives SD correlated positively with small size effect with their husband impotence, premature ejaculation, non-sensuality, dissatisfaction as well as infrequency of sexual intercourse. Also, husbands SD relates positively with small size effect with wives' vaginismus, avoidance, non-sensuality, non-communication and infrequency. However, this effect is of medium size with anorgasmia and sexual dissatisfaction (Table 3). From this study, the higher the subscale score in the husband or wives the higher the corresponding subscales score in their spouse with a small size effect except for avoidance which is of medium size effect. Impotence relates positively with small size effect with their wives vaginismus, anorgasmia, non-sensuality, non-communication and wives infrequency of sexual intercourse. Premature ejaculation also correlates positively with anorgasmia and wives non-communication and infrequency of sex. Husbands non-sensuality also relate with anorgasmia and wife's non-communication. The level of satisfaction of sexual intercourse among the husband correlate positively with anorgasmia and wife's non-sensuality and infrequency of sex. Infrequency of sexual intercourse among the husbands correlates positively with vaginismus and wives non-sensuality (table 3).

**Table 3:** Partial correlation between husbands and wives' sexual dysfunction including the 7 subscales of the GRISS *(N = *179 couples)

		Female sexual dysfunction and its sub-scales							
	**Variables**	**SD**	**VAG**	**ANG**	**AVD**	**NS**	**NC**	**DISS**	**IFQ**

**Male sexual dysfunction and its sub-scales**	Sexual dysfunction (SD)	0.42***	0.25***	0.30***	0.21**	0.26***	0.24**	0.30***	0.23**

	Impotence	0.24**	0.22**	0.23**	0.00	0.17*	0.16*	0.02	0.21**
	Premature ejaculation	0.17*	0.09	0.16*	0.10	0.10	0.24**	0.14	0.16*
	Avoidance (AVD)	0.10	0.04	0.09	0.34***	0.01	0.10	0.08	0.02
	Non-sensuality (NS)	0.22**	0.09	0.21**	-0.02	0.20**	0.17*	0.12	0.03
	Non-communication (NC)	0.08	-0.03	-0.11	0.12	0.01	0.17*	0.08	0.11
	Dissatisfaction (DISS)	0.26***	0.12	0.22**	0.07	0.16*	0.13	0.27***	0.16*
	Infrequency (IFQ)	0.20**	0.19*	0.14	-0.03	0.21**	0.00	0.09	0.23**

*Correlation is significant at the 0.05 level (2-tailed), **Correlation is significant at the 0.01 level (2-tailed), ***Correlation is significant at the 0.001 level (2-tailed). Boldface r = **Pearson product moment correlation coefficient with a medium size (0.30 ≤ r ≥ 0.50) effect, VAG = vaginismus, ANG = anorgasmia**

## Discussion

Sexual dysfunction is defined as disturbances in sexual desire and the psycho-physiological changes that characterize sexual response and cause marked distress as well as interpersonal difficulty [2]. Several social and demographic variables have been identified to influence sexual behaviour and these include gender, race, age, education, marital status and religion. Laumann *et al.*, [22] reported that these variables organize the individuals' pattern of social relations and shape their understanding of the social world thus influencing their sexual behaviour. Therefore, among couples, the occurrence of sexual dysfunction in an interpersonal context will have implications for both partners in the relationship.

The relationship between the quality of sexual function in marriage and the quality of marriage and how the major social and demographic variables (e.g., gender, age, race, education etc) affect marital sex satisfaction have been extensively studied [22-24]. The observed differences in the duration of marriage as a risk factor for sexual dysfunction in husbands and wives in this study could be attributed to the differences in sexual preference or taste and the different ways in which men and women express themselves sexually as related in the study of Laumann *et al.*, [22]. Other researchers have also reported that declines in frequency of marital sex with marital duration is due to the loss of novelty which is often referred to as the "honeymoon effect", meaning that the frequency of marital sex decreases because satisfaction with marital sex declines with marital duration [25,26]. Liu, [27] explained that marital sexual actions between a husband and a wife initially bring about a relatively high level of satisfaction; therefore one can expect sexual activity to be more frequent. As marital sex increases, the level of satisfaction lowers; thus, fewer resources will be allocated to it and consequently the frequency of marital sex declines.

Considerable literature abounds on the relationship between sexual and marital dysfunction [28-32]. The question as to the sort of sexual problems that arise when marriages are disturbed and how sexual problems affect marriages is yet to be answered. Sexual infrequency in husbands was positively linked with vaginismus and non-sensuality in the wives whilst infrequency in the wives' correlated positively with impotence, premature ejaculation and dissatisfaction. Therefore, where sex takes place less often, there could be sexual function problems in either partner which might affect the quality of marriage. Husbands in this study were not only more dissatisfied about their sexual life but also responded more to their spouse's dissatisfaction of sexual acts compared to the wives response to their spouse's dissatisfaction. This implies a reduced level of sexual satisfaction in married couples which is expressed more in the husbands than their wives. This finding agrees well with that of Derogatis *et al.*, [33] who stated that males were more psychologically reactive than women to their partner's sexual dysfunction and attributed it to society's definition of the man's role as the responsible partner regarding satisfaction in sexual relationships. LoPiccolo and Steger, [34] found that males acceptance of their wives sexual pleasure was more important to couples overall satisfaction with the sexual relationship than females acceptance of their husbands sexual pleasure. Frank *et al.*, [35] found males sexual satisfaction to be predicted most strongly by their partner's sexual pleasure and Nowinski *et al.*, [36] reported that for males, the best predictor of sexual behaviour was their estimate of their partner's level of pleasure; for women it was self-reported pleasure.

With the note that sexual and marital dissatisfaction are generally highly related [15], correlational analyses showed that reduced sexual satisfaction in husbands was positively linked with anorgasmia, non-sensuality and infrequency in the wives. Dissatisfaction in the wives' had little impact on the subscales of the husband and this finding agrees well with that of Rust *et al.*, [15] which stated that a woman's dissatisfaction with sexual relationship has little impact on the man's perception of marriage. Donnelly, [11] further demonstrated that lower marital satisfaction is linked with a greater probability of sexual inactivity and separation demonstrating a strong link between marital and sexual satisfaction. This interpretation is further supported by the fact that the sexual dissatisfaction subscales of the GRISS are the only ones in which respondents are asked specifically about their partners rather than about themselves.

The prevalence of sexual dysfunction in husbands (as subjects) (59.2%) and wives (as subjects) (61.5%) are lower than the sexual dysfunction prevalence rates of 65.9% and 72.8% reported in Ghanaian males and females respectively [3,4] in our earlier studies. However, the sexual dysfunction prevalence rates in wives whose husbands have sexual dysfunction (69.8%) and husbands whose wives had sexual dysfunction (67.3%) compares well with the prevalence rates quoted for males [4] and females [3], depicting a greater burden of sexual dysfunction in one spouse when the other is affected and vice versa. The high prevalence rate of sexual dysfunction observed in the wives is however in agreement with the findings of Frank et al., [35] and Spector *et al.*, [37] who have equally reported a high prevalence of sexual dysfunction in females. Most studies have also suggested that sexual dysfunction is more prevalent in women than in men [38,39].

Problems in communication have been noted as a common complaint presented by couples seeking marital therapy [40-42]. Communication has long been considered important to sexual satisfaction and adjustment [43-45]. Non-communication in the wives correlated positively with impotence, premature ejaculation and non-sensuality in the husbands whilst its presence in the husbands had no effect on the subscales of the wives. It could be interpreted that communication deficits, lack of confidence in communicating existing disorders and inhibitions to communication are related to this observation. This could therefore play a central role in the development and maintenance of sexual dysfunction disorders in the various subscales of the GRISS.

Is the sexual problem in each partner due to their own inherent problem or the problem is in response to the problem in their partner? Though the overall results indicated that either direction is plausible as is common causality, further study might be needed to clarify this. It seems likely that sexual dysfunction and a disorder in any of the subscales in one partner might elicit a reduction in sexual function in the other partner which could lead to marital problems.

Some of the limitations of this study include the fact that the study was based on volunteers and self-reported data on socio-demographic information. The GRISS questionnaire has also not been validated in Ghanaians and as such further studies are required to pre-validate the questionnaire among cohorts of Ghanaians.

## Conclusion

The prevalence of sexual dysfunction in married couples is comparable to prevalence rates in the general male and female population and is further worsened by duration of marriage. This could impact significantly on a couple's self-esteem and quality of life thereby causing emotional distress leading to relationship problems. Also spouses' sexual dysfunction is related and as such in married couples, relationship with the spouse should be taken into consideration in treatment processes. It is clear that sexual education does not take place in the family. The national education system should be used more effectively for sexual education. There is a need for more comprehensive surveys, including larger population groups, to be made in order to assess the prevalence of sexual disorders and related factors.

## Competing interests

The authors declare that they have no competing interests.

## Authors' contributions

NA and WKBAO developed the concept and designed the study. NA, WKBAO, EW, LQ and CKG-S administered the questionnaire, analysed and interpreted the data. NA, LQ and CKG-S drafted the manuscript. NA, WKBAO, EWLQ and CKG-S revised the manuscript for intellectual content. All authors read and approved the final manuscript.

## Pre-publication history

The pre-publication history for this paper can be accessed here:

http://www.biomedcentral.com/1471-2490/11/3/prepub
